# Stand‐up exercise training facilitates muscle recovery from disuse atrophy by stimulating myogenic satellite cell proliferation in mice

**DOI:** 10.14814/phy2.12185

**Published:** 2014-11-04

**Authors:** Yuta Itoh, Kimihide Hayakawa, Tomohiro Mori, Nobuhide Agata, Masumi Inoue‐Miyazu, Taro Murakami, Masahiro Sokabe, Keisuke Kawakami

**Affiliations:** 1 Physical and Occupational Therapy Program, Nagoya University Graduate School of Medicine, Nagoya, Japan; 2 Faculty of Rehabilitation Science, Nagoya Gakuin University, Seto, Japan; 3 Mechanobiology Laboratory, Nagoya University Graduate School of Medicine, Nagoya, Japan; 4 Faculty of Health and Medical Sciences, Tokoha University, Hamamatsu, Japan; 5 Aiche Medical College for Physical and Occupational Therapy, Kiyosu, Japan; 6 Faculty of Wellness, Sigakkan University, Ohbu, Japan; 7 Department of Physiology, Nagoya University Graduate School of Medicine, Nagoya, Japan

**Keywords:** 5‐ethynyl‐2’‐deoxyuridine, myogenic satellite cells, myonuclei, operant conditioning, stand‐up exercise

## Abstract

Determining the cellular and molecular recovery processes in inactivity – or unloading –induced atrophied muscles should improve rehabilitation strategies. We assessed the effects of stand‐up exercise (SE) training on the recovery of atrophied skeletal muscles in male mice. Mice were trained to stand up and press an elevated lever in response to a light‐tone cue preceding an electric foot shock and then subjected to tail suspension (TS) for 2 weeks to induce disuse atrophy in hind limb muscles. After release from TS, mice were divided into SE‐trained (SE cues: 25 times per set, two sets per day) and non‐SE‐trained groups. Seven days after the training, average myofiber cross‐sectional area (CSA) of the soleus muscle was significantly greater in the SE‐trained group than in the non‐SE‐trained group (1843 ± 194 *μ*m^2^ vs. 1315 ± 153 *μ*m^2^). Mean soleus muscle CSA in the SE trained group was not different from that in the CON group subjected to neither TS nor SE training (2005 ± 196 *μ*m^2^), indicating that SE training caused nearly complete recovery from muscle atrophy. The number of myonuclei per myofiber was increased by ~60% in the SE‐trained group compared with the non‐SE‐trained and CON groups (0.92 ± 0.03 vs. 0.57 ± 0.03 and 0.56 ± 0.11, respectively). The number of proliferating myonuclei, identified by 5‐ethynyl‐2′‐deoxyuridine staining, increased within the first few days of SE training. Thus, it is highly likely that myogenic satellite cells proliferated rapidly in atrophied muscles in response to SE training and fused with existing myofibers to reestablish muscle mass.

## Abstract (precis)

Stand‐up exercise (SE) training facilitates recovery of myofiber size within 7 days and increases the number of myonuclei during atrophied muscle recovery. This rapid increase in the number of myonuclei derived from myogenic satellite cells may account for the rapid histological changes in atrophied muscles induced by SE training.

## Introduction

Skeletal muscle is a highly plastic tissue that exhibits hypertrophy and atrophy in response to increased and decreased mechanical loading, respectively. In rats, unloading using tail suspension results in soleus muscle atrophy, and mass and size of the muscle decrease by approximately half within 2 weeks. These atrophied muscles recover from\ their normal size after another 2 weeks of reloading (Mitchell and Pavlath 2001; Wang et al. 2006; van der Velden et al. 2007). It was also reported that wheel or treadmill running facilitated the recovery of unloading‐induced atrophied rat soleus and plantaris muscles (Park et al. 1994; Ishihara et al. 2004), which suggested that some muscle activities were involved in shortening the recovery period of atrophied muscle.

It is well established that resistance exercise results in muscle hypertrophy (Gonyea and Ericson 1976; Gonyea 1980; Klitgaard 1988) more effectively than does low‐intensity training, such as wheel or treadmill running. We hypothesized that muscle activity, particularly resistance exercise, would facilitate the recovery of disuse atrophied muscle more efficiently than low‐intensity training. To test this hypothesis, we evaluated the facilitating effects of resistance training on the recovery of atrophied muscle. A loading of ~30% of body weight was reported to be sufficient to induce muscle hypertrophy by resistance training in a normal rodent model (Klitgaard 1988). It was also reported that soleus muscle mass decreased to approximately half of normal muscle after 2 weeks of tail suspension (Mitchell and Pavlath 2001; Knox et al. 2004; Eash et al. 2007; van der Velden et al. 2007), which indicates that loaded force per unit CSA on the atrophied muscle will be approximately twice as large as that on normal muscle if we can ignore body weight loss by tail suspension. In fact, the body weight loss was not larger than 5% in our study here. Therefore, a stand‐up exercise (SE) without additional loading after 2 weeks of tail suspension should bear a mechanical load sufficient enough to induce muscle hypertrophy and was employed as the resistance exercise in this study.

The size of myofibers and the number of myofibers increases during the skeletal muscle hypertrophy induced by the resistance training (Goldspink 1970). In contrast, the size and the number of myofibers decrease during the muscle atrophy induced by unloading (Edgerton et al. 1995; Grounds 2002; Kawashima et al. 2004). Recent studies suggest that satellite cells proliferate and differentiate into myoblasts, which then fuse with exiting myotubes when increasing the size of myofibers during muscle hypertrophy (Rosenblatt et al. 1994; Wang et al. 2006). The increase in the number of myofibers may due to the fusion of the myoblasts into new myofibers (Bischoff 1989; Smith et al. 2001). In this context, it is conceivable that proliferation of satellite cells and differentiation of the satellite cells into myotubes are involved in the recovery of atrophied muscle. However, the cellular and molecular processes have not been elucidated in detail.

In this study, we evaluated the effects of SE training on the recovery of atrophied muscle by tail suspension (TS) in mice. We found that 7 days of SE training was sufficient to induce a nearly full recovery of the soleus muscle that had been atrophied by 2 weeks of TS. The increase in the number of myonuclei in recovered muscle was greater than that in control muscle. An analysis of proliferating myogenic satellite cells during SE suggested that recovery from atrophy was accompanied by an increased number of myonuclei caused by the facilitated fusion of satellite cells with existing muscles.

## Materials and Methods

### Animals

All our experiments were approved by the Animal Care Committee of Nagoya University Graduate School of Medicine (No. 022‐015) and the Animal Care Committee of Nagoya Gakuin University (No. 2007‐007). We followed the guidelines for the care and use of animals established by the Physiological Society of Japan. Ten‐week‐old male ICR mice (SLC Inc., Shizuoka, Japan) were housed at room temperature (25°C) under a 12‐h light–dark cycle and provided food and water ad libitum.

All mice were trained to stand up and press a lever in response to cues that preceded an electric shock. This conditioned behavior (described in detail below) was cued multiple times as a stand‐up exercise. To induce hind limb muscle atrophy, some of these mice were suspended by their tail for 2 weeks so that only their forelimbs could touch the cage bottom (Morey‐Holton and Globus 2002). In brief, a mouse was anesthetized by isoflurane inhalation (1.0%; Isoflu, Abbott Japan Co., Tokyo, Japan) and its tail was attached to a suspension harness. To avoid suffocation due to glossoptosis, tail suspension (TS) was initiated only after a mouse had recovered from anesthesia. A mouse's forelimbs could reach the ground during TS; thus, a mouse was free to move in its cage and could access food and water.

**Figure 1.**
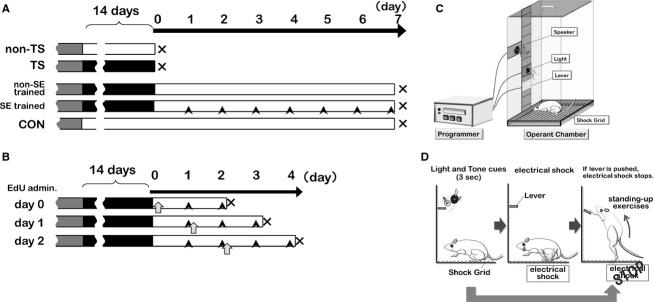
Scheme for intervention times and experimental methods. (A) Treatment protocols for mouse groups: gray, operant conditioning; black, tail suspension (TS); open, normal housing; black arrowhead, stand‐up exercise (SE) training. (B) During SE training, mice were administered 5‐ethynyl‐2’‐deoxyuridine on day 0, 1, or 2 of the SE‐training period (open arrows), and sacrificed 48 h later. (C) Diagram of the operant‐conditioning device. (D) Learning program for stand‐up training. An electrical shock was generated in a shock grid at 3 sec after displaying light and tone cues. The electrical shock was stopped when a mouse pushed the lever. The electrical shock was not used when a mouse pushed the lever in response to the cues before a shock was generated. The mice acquired the stand‐up exercise without an electrical shock after 7 days of learning (100 times/day).

To assess the effects of TS on muscle size, we divided 12 pretrained mice into TS and non‐TS groups (*n* = 6/group; Fig. 1A). Non‐TS mice were housed under the same conditions but were not attached to the TS apparatus. To assess the effects of SE training on atrophied muscles, another 12 TS mice were also divided into two groups (*n* = 6/group; Fig. 1A); one group was subjected to SE training (Fig. 1A arrowhead) after TS (SE trained group), and the other group was fed normally but not exposed to the cues to elicit SE after TS (non‐SE trained group). Finally, we also included a control (CON) group that was not subjected to TS or SE (*n* = 6).

To assess the involvement of myoblast proliferation and fusion in atrophied muscle recovery during SE training, another set of mice was prepared. These mice were administered 5‐ethynyl‐2’‐deoxyuridine (EdU; 25 nmol/g BW, i.p.; Invitrogen, Paisley, UK) at day 0, 1, or 2 of the SE training period (*n* = 6 for each time point, Fig. 1B open arrows). At day 0, mice were administered EdU immediately after being released from TS. For day 1 or 2, mice were administered EdU after SE. Because the half‐life of EdU is ~2 days (Kotogany et al. 2010; Zeng et al. 2010), soleus muscles from all mice were obtained at 48 h after EdU administration (immediately after SE on days 2, 3, and 4 of SE training, respectively) to assess recent de novo DNA synthesis.

### Stand‐up exercise based on operant conditioning

Mice were trained by operant conditioning for 7 days prior to TS using an operant cage (LE2708, Panlab, Bio Research Centre, Nagoya, Japan, Fig. 1C) that was equipped with lights, a speaker, and an electrified bottom. Sound and light cues were generated for 3 sec prior to a benign electric shock (Fig. 1D). The shock could be stopped by pressing an elevated lever on the wall of the operant cage. Mice were trained 100 times each day for 7 days. As a result, the mice learned to stand up in response to the sound and light cues and press the lever to prevent a shock with a success rate of ≥80% (data not shown).

After release from TS, mice in the SE‐trained group were subjected to two sets of cue‐evoked exercise (25 times per set) each day for 7 days, with a 4‐h interval between the sets. The height of the lever was raised so that a mouse's heels did not touch the floor when the mouse touched the lever (distance from the lever to the cage bottom was approximately 8.5–11.0 cm), which resulted in a load to the ankle plantar flexor. At this time, the mouse was marked at the femur third trochanter, lateral condyle, caput fibulae, lateral malleolus, and fifth metatarsal head on the skin and frame advance images were photographed. From these images, different angles were measured. The angle between the femur and fibula was approximately 80° and that between the fibula and fifth metatarsal was approximately 100°. When SE was applied to mice after 2 weeks of TS, the mice could maintain a strike rate at 81 ± 2%, and it was impossible for them to stand up more than 50 times (46.5 ± 2.3 times).

### Immunohistochemistry and histological evaluations

The soleus muscles were harvested from mice in all groups under sodium pentobarbital anesthesia (0.05 mg/g BW, i.p.; Somnopentyl, Kyoritsu Seiyaku, Tokyo, Japan). A soleus muscle was then frozen in isopentane that had been precooled in liquid nitrogen. Subsequently, 8‐*μ*m‐thick frozen transverse sections were prepared through the widest area of the soleus muscle (between 3 mm and 5 mm from the distal tendon). Frozen transverse sections were stained with hematoxylin–eosin (H–E) and photographed under a microscope using a digital camera (Ds‐Ri1, Nikon, Tokyo, Japan). Images were analyzed using Scion Image and ImageJ software to measure the whole‐muscle cross‐sectional area (CSA), myofiber CSA, and the number of myofibers per cross section. To determine the number of myonuclei, the frozen transverse sections were doubly stained with 4’,6‐diamidino‐2‐phenylindole (DAPI; Sigma‐Aldrich, CA) to visualize nuclei and an antidystrophin antibody (Santa Cruz Biotechnology, TX) to visualize the sarcolemma membrane. These slices were fixed with 4% paraformaldehyde for 10 min, washed in PBS, and then blocked with 3% BSA in PBS overnight. After washing in PBS, slices were incubated at 37°C for 60 min with a rabbit antidystrophin polyclonal primary antibody (1:400). After washing, immunolabeled slices were incubated in the dark at 37°C for 45 min with Alexa Fluor^®^ 568‐conjugated goat anti‐rabbit IgG antibody (1:400, Molecular Probes, CA) and DAPI (10 *μ*g/mL).

Slices were photographed under a fluorescent microscope (Nikon ECLIPSE TE300, Tokyo, Japan) and images were analyzed using ImageJ. The numbers of DAPI‐stained nuclei within the antidystrophin‐stained sarcolemma were counted to estimate the mean number of myonuclei per myofiber. The sorting criteria for myonuclei were that if the rim of nuclei was inside the dystrophin ring without overlapping with the ring, those nuclei were considered as myonuclei. In addition, to check whether the number of myonuclei observed in the widest area (see above) of the muscle was also observed throughout the muscle, myonuclei were counted in cross sections taken from each part of the muscle (proximal tip–3 mm, 3–6 mm, 6–9 mm, 9–12 mm, and 12 mm–distal tip of the soleus muscle).

### Newly generated nuclei detection by EdU assay

Nascent nuclei in soleus muscle transverse sections were detected by EdU staining. When EdU staining was performed in conjunction with other immunohistochemical staining, such as antidystrophin staining, the localization of nascent cells could be determined. Sections were fixed for 10 min in 4% paraformaldehyde (all reagents in PBS), washed with 1% BSA, and treated for 20 min with 0.5% Triton^®^ X‐100. After washing with 1% BSA, the sections were blocked with 3% BSA overnight. Thereafter, 500 *μ*L of 1 mmol/L copper (II) sulfate, 200 *μ*L of 100 mmol/L sodium ascorbate, and 300 *μ*L of 1% BSA were mixed with 1 *μ*L of 10 mmol/L Alexa Fluor^®^ 568 azide (Invitrogen, Paisley, UK), and the sections were incubated in this staining solution in the dark at 37°C for 60 min. After washing, slices were stained with an antidystrophin antibody as described above. The number of EdU‐positive myonuclei was determined from these stained sections. In addition, to determine whether the nascent nuclei were in myogenic satellite cells, the sections were triple stained with EdU, DAPI, and anti‐Pax7 monoclonal antibody (Developmental Studies Hybridoma bank, IA), a marker of myogenic satellite cells. After EdU staining, slices were incubated at 37°C for 60 min with a mouse anti‐Pax7 antibody (1:250). After washing, immunolabeled slices were incubated in the dark at 37°C for 45 min with Alexa Fluor^®^ 568‐conjugated goat anti‐mouse IgG antibody (1:400, Molecular Probes, CA) and DAPI.

### Statistical analysis

Results are expressed as means ± standard errors of the mean. Student's *t* tests were used to compare the means of the TS and non‐TS groups. For multiple group comparisons, we used one‐way ANOVA followed by pair‐wise comparisons using a Bonferroni correction. A *P* value of <0.05 was considered significant.

## Results

### Stand‐up exercise training recovers atrophied muscle mass and CSA

**Figure 2.**
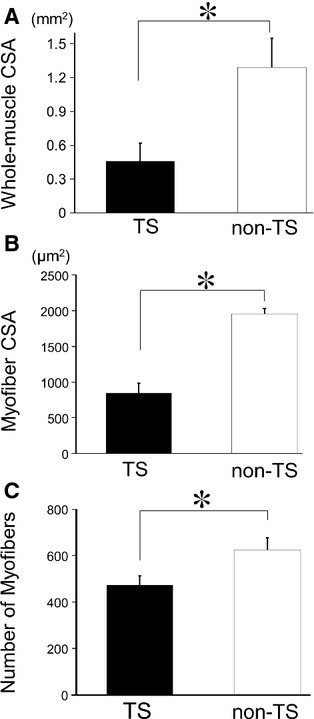
Changes in muscle histology after prolonged tail suspension (TS). (A) Whole‐muscle cross‐sectional area (CSA), (B) myofiber CSA, and (C) the number of myofibers after hind limb unloading using prolonged TS. Whole‐muscle CSA, myofiber CSA, and the number of myofibers were significantly reduced in the TS group compared with the control non‐TS group. Solid bars, TS group; open bars, non‐TS group. Results are means ± SEM's; *n* = 6 per group. *P <**0.05.

We first assessed the histological changes in mouse soleus muscles after 14 days of TS (Fig. 1A). Whole‐soleus muscle CSA was significantly reduced in the TS group compared with that in the non‐TS control group (Fig. 2A). The mean myofiber CSA was also significantly smaller in the TS group compared with that in the non‐TS group (Fig. 2B), as were the numbers of myofibers per muscle (Fig. 2C). Thus, TS had resulted in significant soleus muscle atrophy.

**Figure 3.**
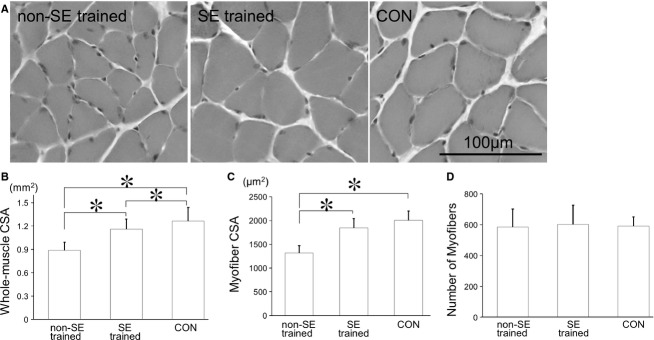
Changes in muscle histology after stand‐up exercise (SE) training. (A) Photomicrographs of muscle samples (hematoxylin–eosin stained) from mice subjected to TS but not SE training (non‐SE‐trained group), subjected to TS and then SE training (SE‐trained group), and control mice (CON). (B) Whole‐muscle CSA, (C) myofiber CSA, and (D) the number of myofibers for these three groups. Myofiber CSA was significantly greater in the SE‐trained group than in the non‐SE‐trained group and approximately the same as in the CON group. There were no significant differences in the numbers of myofibers among these three groups. Results are means ± SEM's; *n* = 6 per group. *P <**0.05.

One group of mice was subjected to SE training for 7 days after their release from TS (SE‐trained group; Fig. 1A). We assessed the effects of SE training on atrophied muscle recovery by evaluating images acquired from soleus muscle transverse sections (Fig. 3A). The average CSA of the whole‐soleus muscle was significantly larger in the SE‐trained group than that in the non‐SE‐trained group, which was subjected to TS but not given exercise cues (Fig. 3B). The average CSA of the whole‐soleus muscle in the SE‐trained group was only slightly smaller than that in the CON group not subjected to TS and SE training, indicating that 7 days of SE training had resulted in nearly complete recovery from soleus muscle disuse atrophy. The whole‐muscle CSA includes the areas of fat, fascia tissues, and interstitial fluid in addition to myofibers. Thus, we measured the CSA of the individual myofibers in order to exclude the area of other tissues.

The mean myofiber CSA in the SE‐trained group was also significantly greater than that in the non‐SE‐trained group, but was not different from that in the CON group (Fig. 3C). In contrast, the average number of myofibers in the SE trained group was not significantly different from that in the non‐SE‐trained and CON groups (Fig. 3D). The decreased number of myofibers observed after TS (see Fig. 2C) suggested that myofibers had recovered within 7 days independent of SE training. In contrast, the increase in whole‐muscle CSA after SE training could have been due to an increase in myofiber CSA rather than an increase in the number of myofibers.

### Stand‐up exercise training increases the number of myonuclei during atrophied muscle recovery

**Figure 4.**
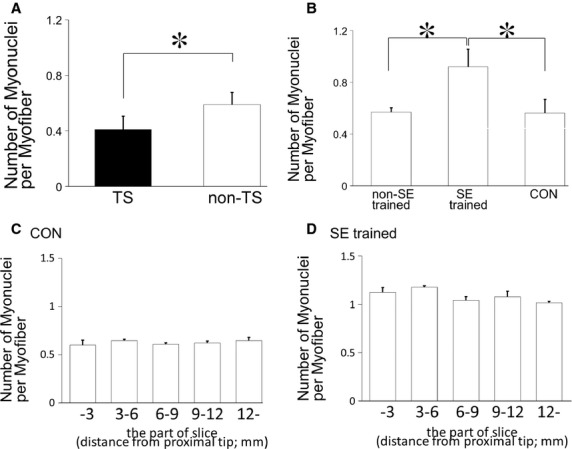
SE‐training effects on numbers of myonuclei in atrophied muscles. (A, B) Numbers of myonuclei per myofiber after TS (A) and SE training (B). There were significantly fewer myonuclei per myofiber in the TS group compared with the control non‐TS group, and there were significantly more myonuclei per myofiber in the SE‐trained group compared with the non‐SE‐trained and CON groups. Results are means ± SEM's; *n* = 6 per group. *P <**0.05. (C, D) The numbers of myonuclei per myofiber were counted in different cross sections of soleus muscles: proximal tip–3 mm, 3–6 mm, 6–9 mm, 9–12 mm, and 12 mm–distal tip. Between these different sections, there were no significant differences in the numbers of myonuclei in the CON (B) and SE trained (C) groups. Results are means ± SEM's; *n* = 3 for each muscle part.

A previous study showed that there was a positive correlation between the size of myofibers and the number of myonuclei per myofiber (Roy et al. 1999). Thus, we investigated changes in the number of myonuclei during SE‐training‐facilitated recovery of atrophied muscles. There were significantly fewer myonuclei per myofiber in the TS group compared with the non‐TS group (Fig. 4A). Bruusgaard et al. (2012) reported that the number of myonuclei in the rat soleus muscle did not decrease after 2 weeks of TS, while the myofiber CSA decreased by 34% in rat soleus muscle. However, this study indicated that the myofiber CSA decreased by 54% and the number of myonuclei decreased by 27% in mouse soleus muscle (Figs 2B, 4A), suggesting that the myofiber CSA decreases in earlier phase of atrophy, then the number of myonuclei decreases in advanced atrophy.

The mean number of myonuclei per myofiber in the non‐SE‐trained group, which exhibited an incomplete recovery of myofiber CSA, was not different from that in the CON group (Fig. 4B). However, the mean number of myonuclei per myofiber in the SE trained group, which exhibited a nearly complete recovery of myofiber CSA, was 1.5 and 1.6 times greater than that in the non‐SE‐trained group and CON group, respectively. An increase in myonuclei was observed in all cross sections of different parts of the soleus muscle (Figs 4C, 4D). These results suggested that a large number of newly proliferated myogenic precursor cells might have fused with atrophied myofibers during SE training to increase CSA and the number of myonuclei.

### Cellular mechanisms of SE‐training‐facilitated recovery from muscle atrophy

**Figure 5.**
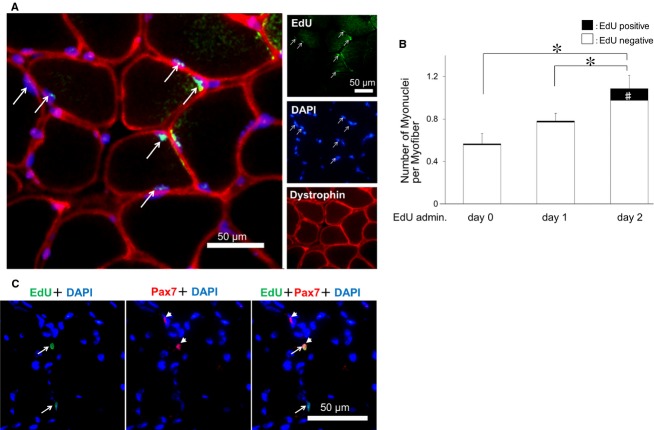
SE‐training‐induced increases in myonuclei are due to the proliferation and fusion of myogenic satellite cells. (A) Typical section stained by 5‐ethynyl‐2’‐deoxyuridine (EdU), antidystrophin, and 4’,6‐diamidino‐2‐phenylindole. (B) Numbers of myonuclei per myofiber and EdU‐positive myonuclei (A, arrow). The number of myonuclei per myofiber was highest in the group administered EdU on day 2 of SE training. *P <**0.05 for total myonuclei counts, ^#^*P *<**0.05 for EdU‐positive myonuclei counts. (C) Photomicrographs of muscle samples stained with an anti‐Pax7 antibody (red, arrowheads), a marker of myogenic satellite cells, from mice subjected to TS and then SE training (SE‐trained group). Immunoreactivity to Pax7 was observed in EdU‐positive nuclei (green, arrows).

To confirm that newly proliferated cells had fused with atrophied myofibers, EdU, a nucleoside analogue used to detect de novo DNA synthesis, was administered to SE trained or non‐SE mice on day 0, 1, or 2 of the SE‐training period (Figs 1B, 5A), and the numbers of EdU‐positive myonuclei were determined 48 h later (on day 2, 3, or 4 of SE training, respectively). There was a significant increase in EdU‐positive myonuclei when EdU was administered on day 2 of SE training. On day 4, EdU‐positive myonuclei accounted for 11.1% of the total myonuclei per myofiber (Fig. 5B). The number of myonuclei on day 4 SE‐trained mice was nearly the same on day 7 SE‐trained mice. (Figs 4B, 5B). These results suggested that myogenic precursor cells had begun to proliferate as early as 2 days after the SE‐training onset, and then fused with existing myofibers within 2 days (between 2 and 4 days of SE training). The EdU‐positive myonuclei was hardly detected in the non‐SE‐trained mice at any time point (0.0003, 0.01, 0.02% on day 2, 3, or 4, respectively).

Immunoreactivity for Pax7, a marker of muscle myogenic satellite cells, was also observed in EdU‐positive cells at day 4 of SE training (Fig. 5C), which suggested that activation of quiescent myogenic satellite cells and proliferation of these cells were initiated in atrophied muscles by SE training. More EdU‐positive myonuclei were observed in mice that were administered EdU on day 2 of SE training compared with mice that were administered EdU on day 0 or 1 of SE training, which suggested that myogenic satellite cell proliferation had begun on days 2–4 of SE training, based on the half‐life of EdU (approximately 48 h).

## Discussion

### Stand‐up exercise training facilitates the recovery of atrophied muscle

In this study, we demonstrated that SE training after disuse atrophy facilitated the recovery of atrophied muscle in mice. At 14 days after TS, soleus muscle wet weight and CSA had decreased to 75% (data not shown) and 40% (Fig. 2A) of the control, respectively, which agreed with the results of previous studies on the effects of prolonged TS (Musacchia et al. 1980; Morey‐Holton and Globus 2002; Knox et al. 2004; Eash et al. 2007). The mean myofiber CSA decreased to 43% and the number of myofibers decreased to 75% of the control immediately after TS, which indicated that the decreased weight of the atrophied soleus muscle was due to a decrease in both the size and number of myofibers.

When these mice were fed normally and without SE training after TS, their mean myofiber CSA did not recover to the level of CON group by 7 days. In contrast, the mean myofiber CSA of mice that were subjected to SE training recovered to nearly the same level of CON group within 7 days. The short‐term exercise training (less than 60 min/day) on normal muscle require 8–16 weeks for muscle hypertrophy (Lowe and Alway 2002), this period is quite longer than that of the exercise‐facilitated recovery of atrophied muscle. This suggests that the increase in myofiber CSA in atrophied muscle by exercise training may be endowed with the early cellular response(s) that is different from the hypertrophic response(s) in the normal muscle by resistance training. It was previously reported that a mechanical load exceeding 30% of the normal body weight was enough to induce hypertrophy in normal muscles (Gonyea and Ericson 1976; Gonyea 1980; Klitgaard 1988). Thus, SE without additional weight loading appeared to have sufficient intensity to induce muscle hypertrophy in atrophied muscles, because the force per unit CSA loaded on the atrophied muscle will be approximately twice as large as that on normal muscle due to the reduced CSA of the atrophied muscle by approximately half of the normal muscle in CON group.

An increase in the muscle belly thickness was associated with an increase in the actual numbers of myofibers in several studies (Gonyea 1980; Tamaki et al. 1992; Antonio and Gonyea 1993a,b), but not in others (Gollnick et al. 1981, 1983; Paul and Rosenthal 2002). In this study, the number of myofibers decreased immediately after TS, but returned to the normal level after 7 days independently of SE training (Fig 3D); this indicated that low‐intensity stimulation, such as normal cage activity, was sufficient to increase the number of myofibers. This also suggested that low‐intensity stimulation was sufficient to activate quiescent myogenic satellite cells and to induce their proliferation and fusion to form new myofibers, whereas greater intensity stimulation is required for the rapid recovery of myofiber CSA.

### Increase in the number of myonuclei during facilitated recovery from muscle atrophy

In previous studies, resistance exercise for normal muscle increased their contractile strength during the first 20 days, which reflected an increased firing frequency (Van Cutsem et al. 1998; Patten et al. 2001) and synchronization of motor units (Milner‐Brown et al. 1975; Semmler and Nordstrom 1998). Myofiber hypertrophy of normal muscle was observed at the histological level after 8–16 weeks of exercise training (Gonyea and Ericson 1976; Gonyea 1980; Klitgaard 1988; Narici et al. 1989; Ploutz et al. 1994; Lowe and Alway 2002). In this study, major histological changes were observed earlier, even on day 7 after the onset of SE training (Fig. 3). This suggests that recovery from disuse atrophy facilitated by SE training could have been mediated by much more rapid responses in atrophied muscle compared with the normal muscle hypertrophy induced by resistance exercise.

It was reported that the number of myogenic precursor cells increased by 4‐ to 5‐fold prior to an increase in the number of myonuclei in normal muscle hypertrophy (Smith et al. 2001). In general, myonuclei are believed not to multiply after their terminal differentiation in mammals (Stockdale 1971). In addition, there was no increase in the number of myonuclei when myogenic satellite cells were inactivated by irradiation (Rosenblatt et al. 1994). These results suggest that high‐intensity stimulation initiates proliferation of myoblasts and their fusion to existing myofibers. Thus, it is conceivable that the increase in the number of myonuclei induced by SE training observed in this study was due to the proliferation of myogenic satellite cells and the fusion of differentiated myogenic satellite cells to existing myofibers.

In another study, the number of myonuclei increased on 8–14 days after muscle overload by ablating synergist muscles in normal rats (Rosenblatt et al. 1994; Bruusgaard et al. 2010). These models were functional overload model by partially ablating of the synergist muscle; therefore, the target muscle was stimulated all day long. In our study, the number of myonuclei in atrophied muscles started to increase as early as on day 4 after the onset of SE training (Fig. 5B), suggesting that the increase in the myonuclei number in atrophied muscle induced by exercise training occurs much earlier than in the exercise‐induced hypertrophy in normal muscle. This rapid increase in the number of myonuclei derived from myogenic satellite cells may account for the rapid histological changes in atrophied muscles induced by SE training. A recent study showed that myogenic satellite cell null mice (Jackson et al. 2012) still had an increase in the size of myofibers by reloading, which indicated that myogenic satellite cells are not indispensable for the recovery of the size of myofibers from unloading‐induced muscle atrophy. In our study, the increase in the number of myonulcei was greater in SE‐trained myofibers and their average CSA recovered earlier compared with non‐SE‐trained mice. These results suggest that CSA of myofibers gradually recovers from disuse atrophy without fusion of myogenic cells with preexisiting myofibers, although such fusion induced by mechanical loading, including SE, effectively facilitates the recovery of myofiber size from muscle atrophy.

In summary, we evaluated the effects of SE training on the recovery from muscle atrophy in mice using an integrated analysis of whole‐muscle CSA, myofiber CSA, the numbers of myonuclei, and the origin of these myonuclei. SE training facilitated the recovery of whole‐muscle and myofiber CSA and increased the number of myonuclei. The relatively rapid increase in the number of myonuclei observed in this study may presumably be derived from newly proliferated and fused myogenic satellite cells. The increased myonuclei may, in turn, have contributed to the rapid recovery of whole muscle and myofiber CSA. In contrast, the recovery of myofiber number was independent of SE training. Determining the underlying molecular mechanisms involved in these responses remains to be solved.

Collectively, above results indicate that resistance training would be very beneficial if it is introduced properly based on the history and degree of atrophy. Basic study like present one must be quite helpful to validate the optimal protocol, including period, timing, and intensity of resistant training, for treating patients under rehabilitation.

## Acknowledgments

The authors thank Dr. Hiroyuki Soga at the Kinjo University for valuable comments.

## Conflict of interest

None declared.
